# Interferon-β-related tumefactive brain lesion in a Caucasian patient with neuromyelitis optica and clinical stabilization with tocilizumab

**DOI:** 10.1186/s12883-014-0247-3

**Published:** 2014-12-17

**Authors:** Jens Harmel, Marius Ringelstein, Jens Ingwersen, Christian Mathys, Norbert Goebels, Hans-Peter Hartung, Sven Jarius, Orhan Aktas

**Affiliations:** Department of Neurology, Medical Faculty, Heinrich-Heine-University, Moorenstr. 5, 40225 Düsseldorf, Germany; Department of Diagnostic and Interventional Radiology, Medical Faculty, Heinrich-Heine-University Düsseldorf, Moorenstr. 5, 40225 Düsseldorf, Germany; Molecular Neuroimmunology, Department of Neurology, University of Heidelberg, Otto Meyerhof Center, Im Neuenheimer Feld 350, 69120 Heidelberg, Germany

**Keywords:** Neuromyelitis optica, Aquaporin-4 antibodies (NMO-IgG), Interferon-beta, Tocilizumab, Interleukin-6, Rituximab, Azathioprine, Methylprednisolone, Tumefactive brain lesions, Multiple sclerosis

## Abstract

**Background:**

Neuromyelitis optica (NMO) is a severely disabling inflammatory disorder of the central nervous system and is often misdiagnosed as multiple sclerosis (MS). There is increasing evidence that treatment options shown to be beneficial in MS, including interferon-β (IFN-β), are detrimental in NMO.

**Case presentation:**

We here report the first Caucasian patient with aquaporin 4 (AQP4) antibody (NMO-IgG)-seropositive NMO presenting with a tumefactive brain lesion on treatment with IFN-β. Disease started with relapsing optic neuritis and an episode of longitudinally extensive transverse myelitis (LETM) in the absence of any brain MRI lesions or cerebrospinal fluid-restricted oligoclonal bands. After initial misdiagnosis of multiple sclerosis (MS) the patient received subcutaneous IFN-β1b and, subsequently, subcutaneous IFN-β1a therapy for several years. Under this treatment, the patient showed persisting relapse activity and finally presented with a severe episode of subacute aphasia and right-sided hemiparesis due to a large T2 hyperintensive tumefactive lesion of the left brain hemisphere and a smaller T2 lesion on the right side. Despite rituximab therapy two further LETM episodes occurred, resulting in severe neurological deficits. Therapeutic blockade of the interleukin (IL)-6 signalling pathway by tocilizumab was initiated, followed by clinical and radiological stabilization.

**Conclusion:**

Our case (i) illustrates the relevance of correctly distinguishing NMO and MS since these disorders differ markedly in their responsiveness to immunomodulatory and -suppressive therapies; (ii) confirms and extends a previous report describing the development of tumefactive brain lesions under IFN-β therapy in two Asian NMO patients; and (iii) suggests tocilizumab as a promising therapeutic alternative in highly active NMO disease courses.

## Background

Interferon-β (IFN-β) is one of the established first-line therapies for patients with relapsing-remitting multiple sclerosis (MS). While the efficacy of IFN-β in MS is widely accepted, the possible benefit in non-classical MS variants has been disputed. This is the case for neuromyelitis optica (NMO/Devic’s syndrome), considered an MS subtype until the discovery of the anti-aquaporin 4 (AQP4) antibodies (AQP4-Ab) [1,2]. In both Asian and European NMO patients, IFN-β failed to show therapeutic efficacy [3-5]. Of note, Shimizu and colleagues recently reported two Asian patients with NMO spectrum disease (NMOSD) who developed extensive brain lesions following IFN-β therapy [6]. Here we report the case of an AQP4 Ab-seropositive NMO patient of Caucasian descent presenting with a tumefactive lesion on IFN-β treatment. Of note, our patient did not respond to B cell depletion by the monoclonal anti-CD20 antibody rituximab but improved upon blockade of the interleukin (IL)-6 pathway using tocilizumab.

## Case Presentation

**Figure 1:**
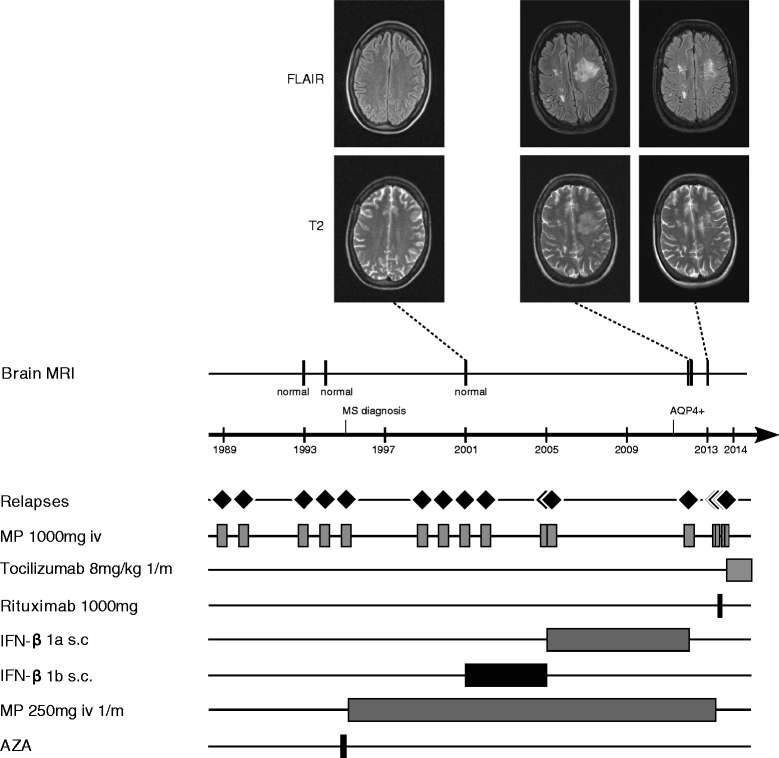
**Disease course, MRI scans and therapy in a patient with AQP4-Ab-positive NMO.** The figure depicts clinical relapses (diamonds), MRI findings (T2, FLAIR), and therapy data (methyl prednisolone (MP) 1000 mg i.v. 3–5 daily infusions; tocilizumab 8 mg/kg bodyweight once a month; rituximab 1000 mg i.v. (single infusion), IFN (interferon)-β1a 44 μg s.c. three times a week (Rebif®); IFN-β1b s.c. daily (Betaferon®, Betaseron®); methylpredisolone 250 mg i.v. once per month; azathioprine (AZA)). No relapses have occurred during therapy with tocilizumab (follow-up: 12 months). AQP: aquaporin, AZA: azathioprine, FLAIR: fluid attenuated inversion recovery, IFN: interferon, MP: methyl prednisolone, MRI: magnetic resonance imaging, MS: multiple sclerosis, NMO: neuromyelitis optica.

A 47-year old Caucasian female patient had been diagnosed with MS in 1995 in an external hospital after four episodes of optic neuritis (ON) since 1989 and an episode of longitudinally extensive transverse myelitis (LETM) in 1995 (Figure 1). Cerebrospinal fluid (CSF)-restricted oligoclonal bands (OCBs) were repeatedly negative and brain MRI scans did not show any abnormalities (e.g. MRI performed in 2001, Figure 1). After an initial two months azathioprine treatment in 1995, which had to be discontinued because of side effects, repeated monthly intravenous (iv) steroid pulse treatments with 250 mg methylprednisolone (MP) were initiated and continued until April 2013. In 2001, after three further episodes of ON, subcutaneous IFN-β1b medication was added to the ongoing steroid pulse treatment. In 2005, subcutaneous IFN-β1b was switched to subcutaneous IFN-β1a (3 × 44 μg/week). During IFN-β therapy the patient still suffered from further relapses, most of them occurring between the years 2001 and 2005 (July 2002, February 2005, and April 2005). She recovered only partially from the relapses and accumulated fixed neurological deficits such as amaurosis of the right eye and impaired walking with an expanded disability status scale (EDSS) score of 4.0 in January 2012. Of note, there was no chronic progression between the relapses. In 2011 persisting disease activity prompted testing for serum AQP4 antibodies (by a cell-based assay; CBA [7]) which were found positive and confirmed the diagnosis of seropositive NMO. Following the patient’s wishes and in light of the reduced relapse rate since 2005 therapy with subcutaneous IFN-β1a was continued, together with the regular monthly steroid therapy (250 mg MP each) established since 1995. In January 2012, she suffered from a subacute aphasia and right sided hemiparesis and presented to our department. MRI imaging showed a large T2 hyperintensive, tumefactive lesion in the prefrontal white matter of the superior and middle frontal gyrus of the left hemisphere and smaller T2 lesions on the right side without clear evidence of a disturbed blood brain barrier (Figure 1). MR spectroscopy showed an unchanged choline peak and thus did not give a hint to a malignant process. Analysis of CSF was unremarkable with still no quantitative evidence for intrathecal immunoglobulin (Ig) synthesis or OCBs and a normal CSF white cell count. AQP4-Ab serum titre was 1:1000 (CBA). Furthermore, a time-of-flight MR-angiography showed no signs of vasculitis, and laboratory work-up revealed no clinical or serological evidence for connective tissue diseases. The patient received a high-dose methylprednisolone iv pulse and clinically improved. The follow-up MRI one month later showed a mild regression of the large T2 hyperintense lesion. We stopped IFN-β therapy and continued the already existing monthly steroid pulses (250 mg MP each) as the patient declined any additional medication at that time. However, two attacks of myelitis occurred in March 2013 and April 2013. Follow-up AQP4-Ab testing in April 2013 confirmed seropositivity (1:1000; CBA). The patient was treated with high-dose steroid iv pulse therapy and rescue plasmapheresis. Thereafter our patient agreed to an adaptation of therapy and the monthly steroid infusions were discontinued without tapering.

We initiated treatment with rituximab (single intravenous infusion of 1000 mg) in May 2013 and transferred our patient to a rehabilitation centre. However, further severe LETM attacks occurred shortly thereafter in June and August 2013. The patient was re-admitted to our department in September 2013 and showed persistently increased AQP4-Ab titres (1:3200, CBA). Absolute B cell counts were normal (68/μl) while relative proportion was decreased (3% of all lymphocytes; reference value 5-24%). This relapse was associated with a urinary tract infection (without systemic signs of inflammation, i.e. without leucocytosis, fever or elevated C-reactive protein (CRP) levels) which we successfully treated according to local guidelines. In light of the patient’s severe clinical deficits (EDSS of 9.0) we applied another iv steroid pulse and decided to switch to tocilizumab, a humanized monoclonal anti-IL-6-receptor antibody. Monthly infusions of tocilizumab with 8 mg/kg bodyweight were followed by clinical stabilization as no further relapses occurred for the following 12 months (see clinical and MRI follow-up after withdrawal of IFN-β in Figure 1). We also observed a slight clinical improvement, as reflected by an EDSS of 8.0. Of note, serum AQP4 antibodies were still detectable at high titre at last follow-up in October 2014 (1:3200, CBA).

## Conclusions

To our knowledge, we here present the first Caucasian NMO patient who developed a tumefactive brain lesion on long-term IFN-β treatment. Our case confirms and extends similar findings in two Asian NMOSD patients recently reported by Shimizu and colleagues [6]. Obviously the development of a tumefactive brain lesion during IFN-β therapy of NMO is not linked to a specific ethnicity. Moreover, in line with results obtained previously in other cohorts, our patient experienced numerous relapses under IFN-β therapy. Why NMO patients fail to respond to IFN-β remains so far unclear. Possible explanations consider the complex immunomodulatory effects of IFN-β, including the upregulation of proinflammatory cytokines observed in MS [8]. Since in NMOSD higher CNS levels of IL-17 are observed than in MS [9], it may be speculated that IL-17 biology may play a role in NMO unresponsiveness to IFN-β. Moreover, a recent study in NMO patients could link serum levels of IFN-α, a type I interferon closely related to IFN-β, with clinical disease activity and severity [10]. Independent experimental work demonstrated that NMO-IgG-mediated astrocyte damage is reduced in the CNS of type I interferon receptor deficient mice, suggesting that type I interferon signalling contributes to the pathogenesis of neuromyelitis optica [11].

Of note, administration of a single intravenous infusion of 1000 mg rituximab was followed by further relapses just one and three months later. While CD19 counts were not determined directly after rituximab application, an investigation four months after rituximab dosing showed normal absolute B cell counts and persistently increased AQP4-Ab levels. We may speculate that our patient belongs to individuals characterized by an early repopulation of the B cell compartment following rituximab therapy [12].

Our case adds preliminary evidence in favour of a possible beneficial effect of tocilizumab in NMO, even following treatment with rituximab [13-15]. However, considering that NMO is often characterized by very long periods free of any relapses, the suggested success of tocilizumab in our patient needs to be followed up. Moreover, more studies, in particular independent controlled trials, are required to confirm the efficacy of tocilizumab in NMO suggested by this case report and previous ones [13-16]. The demonstration of significantly increased levels of IL-6 in comparison to MS in the CSF and serum of patients with NMO [17,18], particularly in AQP4 Ab-seropositive patients with severe disease activity [18], provides a rationale for the use of this drug in NMO. In an *ex vivo* experimental model of NMO spinal cord slice cultures exposed to NMO-IgG and complement showed a marked loss of AQP4 and myelin which was enhanced by adding IL-6 [19]. Moreover, Chihara and colleagues described a specific IL-6-dependent B lymphocyte subpopulation in the peripheral blood and CSF of NMO patients: these CD19^+^ CD27^+^ CD38^+^ CD180^+^ B cells were found to produce AQP4 antibodies and showed enhanced survival as well as AQP4 antibody secretion in the presence of IL-6, whereas blockage of the IL-6 receptor signalling by an anti-IL-6R antibody shortened their survival *in vitro* [20].

Our report illustrates the importance of correctly diagnosing NMO and MS to avoid mistreatment with potentially severe or even fatal consequences. Two recent studies have revealed that up to 30% of patients with NMO were falsely diagnosed with MS [21,22] and, in consequence, often treated with drugs not effective in NMO. While AQP4-IgG serum testing is certainly the most important differential diagnostic measure, other investigations such as lumbar puncture or brain and spinal cord MRI are important as well. In the present case, the diagnosis of MS was made in the absence of CSF-restricted OCBs. OCB negativity is extremely rare in MS (~2-5%) and should prompt physicians to question that diagnosis; by contrast, around 70% of patients with AQP4-IgG-positive NMOSD lack OCBs [22-24]. Normal CSF/serum concentration quotient values of IgG (QIgG) in NMOSD are especially common during periods of clinical remission [23]. Similarly, a persistently normal brain MRI as observed in our patient should warn against the diagnosis of MS [25]. Brain lesions are absent in most patients with NMO at disease onset, but may occur later in the disease and may even meet MRI criteria for MS [23,26]. Of note, for Asian NMO patients, independent case reports described the occurrence of hemispheric brain lesions even without association to IFN-β therapy [27-29], while we are not aware of such observations for NMO patients of other ethnic background. Finally, predominantly central, longitudinally extensive spinal cord lesions extending over more than three vertebral segments are extremely rare in MS while their presence is highly suggestive of NMOSD [30].

Our case supports the conclusion that the development of tumefactive brain lesions under IFN-β therapy for suspected MS should prompt considering NMOSD as the underlying disease, obviously not only in Asian but also in Caucasian patients. Moreover, our report underlines – in accordance with previous reports and recommendations [31] – the need to treat MS and NMO, two conditions with substantial differences in pathogenesis [32,33] differentially and, in particular, to avoid treatment with IFN-β in patients with NMO. Our report adds to previous evidence indicating a potential treatment effect of tocilizumab, an IL-6 signalling pathway blocker in NMO therapy.

## Consent

Written informed consent was obtained from the patient for publication of this case report and any accompanying images. A copy of the written consent is available for review by the Editor of this journal.
